# DSGOST regulates resistance via activation of autophagy in gastric cancer

**DOI:** 10.1038/s41419-018-0658-y

**Published:** 2018-05-29

**Authors:** Tae Woo Kim, Seon Young Lee, Mia Kim, Chunhoo Cheon, Bo-Hyoung Jang, Yong Cheol Shin, Seong-Gyu Ko

**Affiliations:** 1 Department of Preventive Medicine, College of Korean Medicine, Kyung Hee University, Seoul, Korea; 2 Department of Cardiovascular and Neurologic Disease (Stroke center), College of Korean Medicine, Kyung Hee University, Seoul, Korea

## Abstract

Danggui-Sayuk-Ga-Osuyu-Saenggang-Tang (DSGOST in Korean, Danggui-Sini-Jia-Wuzhuyu-Shengian-Tang in Chinese, and Tokishigyakukagoshuyushokyoto (TJ-38) in Japanese), a well-known traditional Korean/Chinese/Japanese medicine, has long been used to treat vascular diseases such as Raynaud’s phenomenon (RP). However, anticancer effect of DSGOST remains elusive. In this study, we checked if DSGOST has an anticancer effect against gastric cancer cells, and investigated the mechanisms underlying DSGOST resistance. Moreover, DSGOST regulates chemoresistance in cisplatin-treated gastric cancer cells. Interestingly, DSGOST treatment induced the accumulation of GFP-LC3 puncta and increased the level of autophagy markers, such as LC3-II, ATG5, and Beclin-1, indicating activated autophagy. Furthermore, DSGOST could activate epithelial-to-mesenchymal transition (EMT) and exosomes via induction of autophagy. DSGOST in combination with TGFβ also induced autophagy and EMT. However, autophagy inhibition induces DSGOST-mediated cell death in gastric cancer cells. In addition, autophagy inhibition blocks the activation of DSGOST-mediated EMT markers including N-cadherin, Snail, Slug, vimentin, β-catenin, p-Smad2, and p-Smad3. Taken together, these findings indicated that prosurvival autophagy was one of the mechanisms involved in the resistance of gastric cancer to DSGOST. Targeting the inhibition of autophagy could be an effective therapeutic approach to overcome resistance to DSGOST in gastric cancer.

## Introduction

Herbal medicines have long been used in East-Asian countries, including Korea, China, and Japan as alternative therapies for symptomatic relief from various disease^1,2^. Danggui-Sayuk-Ga-Osuyu-Saenggang-tang (DSGOST) is a traditional Korean herbal medicine that has been handed down to us from ancient Korean history, and is similar to the traditional Chinese herbal medicine Danggui-Sini-Jia-Wuzhuyu-Shengjian-Tang and the traditional Japanese herbal medicine Tokishigyakukagoshuyushokyoto (TJ-38)^3–5^. DSGOST is used in the therapy of patients having Raynaud’s phenomenon (RP) who suffer from cold in the extremities^6^. RP is episodic ischemia that occurs in response to cold environment exposure, and patients with RP suffer cold hypersensitivity on hands and feet^7^. In the hands of patients with RP, localized cooling increases the adrenergic neurotransmitter mechanism^8^. In patients with RP, nervous system initiates exaggerated vasoconstriction in response to cooling^9^. The vasodilatory and anticoagulant ingredients of TJ-38 markedly relieved peripheral coldness^10,11^. In addition, cold-induced vasoconstriction occurs due to the activation and translocation of adrenoceptor alpha 2C (ADRA2C), and cold condition induces Rho kinase activation^12^. In our previous study, we suggested that DSGOST blocks cold-induced Rho A activation and the endothelin-1 pathway in vascular smooth muscle and endothelial cells^13^.

RP is a known adverse effect of cancer chemotherapy^14^. it could be caused by various anticancer drugs, including cisplatin, vinblastine, and bleomycin^15,16^. A recent report indicates that DSGOST blocks tumor growth by suppressing angiogenesis in pancreatic cancer, and this report also suggests that DSGOST has potential use in effectively reducing the tumor volume during cancer therapy^17^. DSGOST ingredients suggest a possibility for its use in anticancer therapy against many cancer types^18–25^. Various components of DSGOST have been studied for their anticancer effects, including cell death, apoptosis, and antiproliferation, and from the results, DSGOST shows a potential for use in cancer therapy.

Autophagy is a self-degradation process that occurs during starvation and growth deprivation and under stress conditions^26^. Autophagy has a dual role in promoting cell death and survival in cancer^27,28^. Some reports suggest that autophagy regulates chemoresistance in various cancer types^29,30^. Cisplatin and 5-fluorouracil (5-FU) induce cell death in various cancer cells; however, chemoresistant cancer cell lines promote a cell survival mechanism via activation of autophagy, and autophagy inhibition changes to therapeutic effect from chemoresistant to chemosensitive^31^. In addition, cisplatin-mediated chemoresistance induced a pro-survival process via the activation of autophagy in nasopharyngeal carcinoma and displayed epithelial−mesenchymal transition (EMT) including the upregulation of vimentin^32^. Chemoresistance through autophagy activation acquires EMT phenotype, and crosstalk between EMT and autophagy suggests a new direction for chemotherapeutic strategy^33^.

Therefore, we identified the dual effect of DSGOST and cisplatin for anticancer therapy in gastric cancer and studied the mechanisms underlying the resistance of gastric cancer to DSGOST. We also suggested that DSGOST-mediated gastric cancer cells acquire chemoresistance via autophagy induction and undergo EMT but autophagy inhibition causes DSGOST-induced cell death in gastric cancer.

## Results

### DSGOST regulates resistance in gastric cancer

**Fig. 1:**
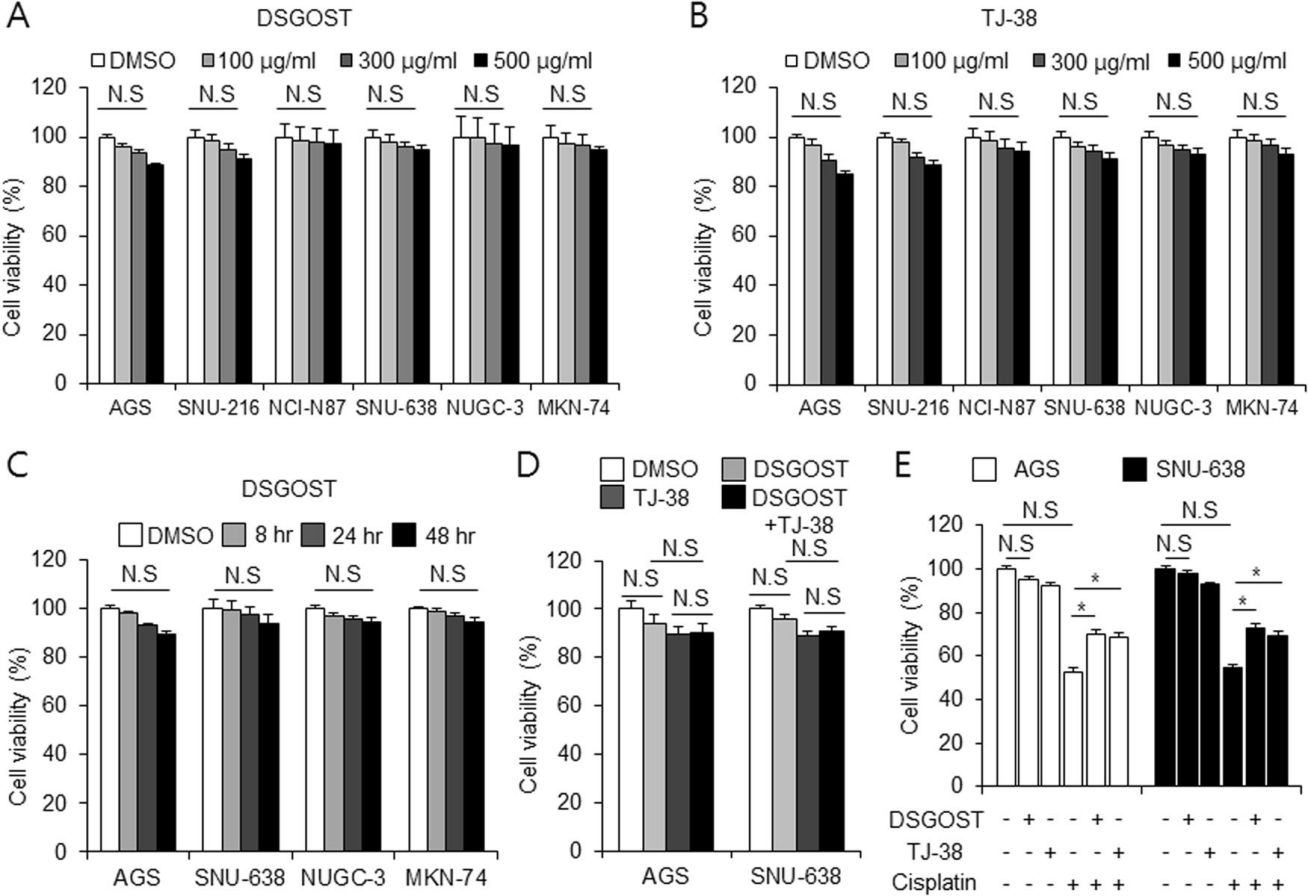
Effect of DSGOST, TJ-38, and cisplatin in gastric cancer. **a**, **b** Cell viability of DSGOST and TJ-38 in gastric cancer cells measured using WST-1 on 96-well plates, and DSGOST and TJ-38 were treated in a dose-dependent manner (100, 300, and 500 μg/mL; 24 h). **c** Cell viability of DSGOST in gastric cancer cells measured using WST-1 on 96-well plates, and DSGOST was treated in a time-dependent manner (500 μg/mL; 8, 24, and 48 h) **d** Cell viability of DSGOST (500 μg/mL, 24 h) and TJ-38 (500 μg/mL, 24 h) using WST-1 in gastric cancer cells. **e** Cell viability of DSGOST (500 μg/mL, 24 h) or TJ-38 (500 μg/mL, 24 h) in combination with cisplatin (5 µM, 24 h) measured in gastric cancer cells using WST-1. Cell viability of DMSO-treated control cells was set at 100%; *p < 0.05

To check the effect of DSGOST on various gastric cancer cells, we performed cell viability assay. DSGOST did not inhibit the cell viability of these cells in a dose-dependent manner (100, 300, and 500 µg/mL, 24 h) (Fig. 1a). To investigate the effect of TJ-38, cell viability assay was performed in a dose-dependent manner (100, 300, and 500 µg/mL, 24 h) (Fig. 1b). TJ-38 did not affect cell viability of gastric cancer cells. We checked the effect of DSGOST in a time-dependent manner (0, 8, 24, 48 h) on gastric cancer cell lines, and identified no effect on cell viability (Fig. 1c). Furthermore, treatment with DSGOST (500 µg/mL, 24 h) plus TJ-38 (500 µg/mL, 24 h) did not affect cell viability in gastric cancer cells (Fig. 1d). Next, we examined the effect of DSGOST (500 µg/mL, 24 h) or TJ-38 (500 µg/mL, 24 h) in combination with cisplatin (5 µM, 24 h) (Fig. 1e). The combination of cisplatin with DSGOST or TJ-38 recovered cell viability of gastric cancer cells to a greater extent than cisplatin alone did. These findings suggest that DSGOST and TJ-38 induce chemoresistance in cisplatin-treated gastric cancer cells.

### DSGOST induces autophagy in gastric cancer

**Fig. 2:**
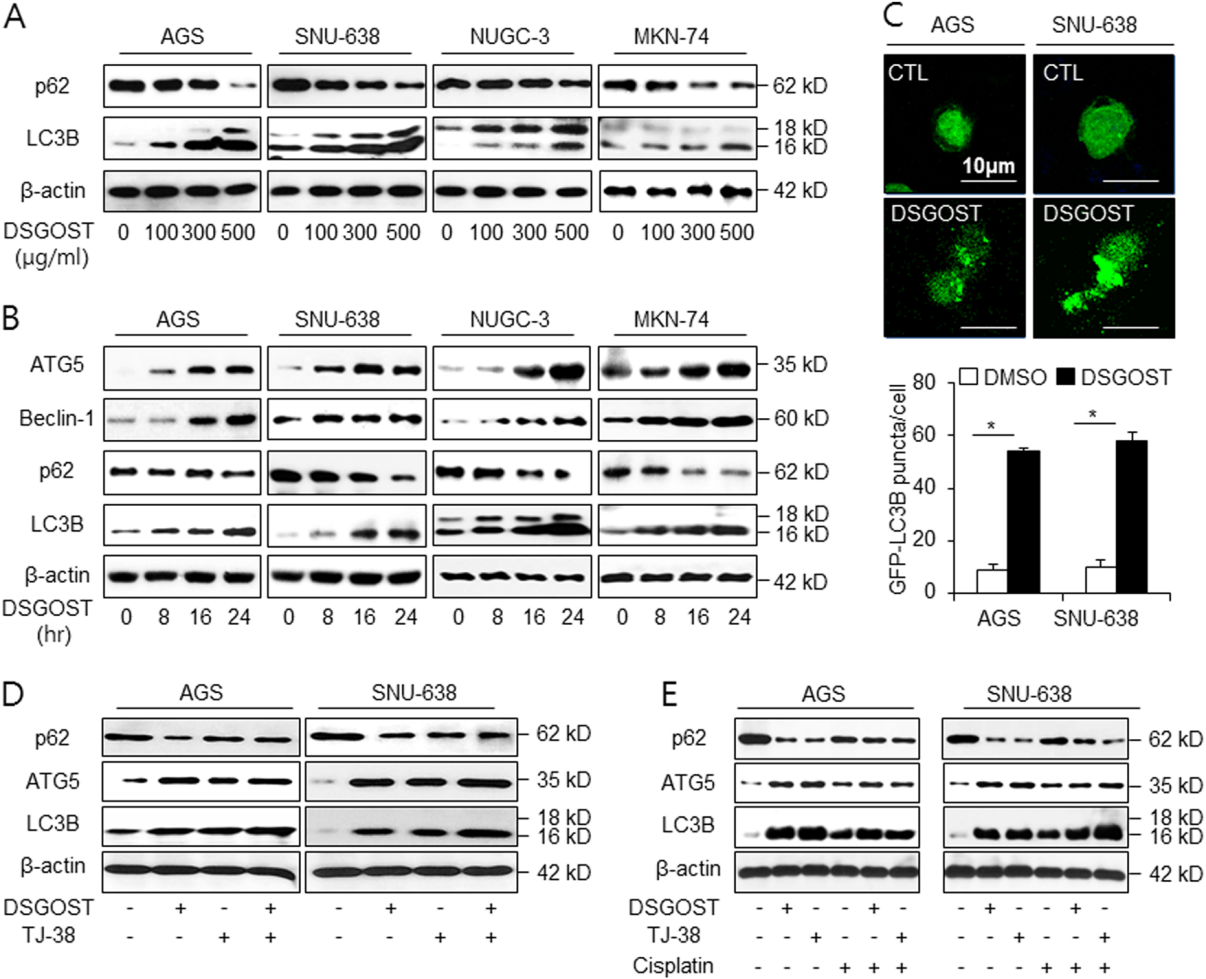
DSGOST activates autophagy in gastric cancer cell lines. **a**, **b** AGS and SNU-638 were treated with DSGOST in a dose- (100, 300, and 500 μg/mL; 24 h) and a time-dependent manner (8, 16, and 24 h; 500 μg/mL), and the control cells were treated with DMSO. Sampling of total lysates was conducted by western blot assay to identify the activation of autophagy markers in a DSGOST dose- and time-dependent manner. **c** AGS and SNU-638 cells transfected by the pEGFP-LC3 vector were treated with DMSO or DSGOST (500 μg/mL) for 8 h. Fluorescence microscopy analysis calculated by puncta of LC3B staining. The graph indicates the number of cells with GFP-LC3B puncta; *p < 0.05. **d** Western blot assay of LC3B, ATG5, and p62 level in DSGOST (500 μg/mL, 24 h) and/or TJ-38 (500 μg/mL, 24 h)-treated AGS and SNU-638 cells. **e** After AGS and SNU-638 cells were treated with DSGOST (500 μg/mL) or TJ-38 (500 μg/mL) in combination with cisplatin (5 µM) for 24 h, protein samples were loaded to perform western blotting for LC3B, ATG5, and p62. β-actin was used as the protein loading control

Increasing reports suggest that autophagy plays an important role for resistance in cancer^34^. LC3-I is converted to LC3-II via lipidation during autophagy^35^. p62 binds directly to LC3 and is a useful marker of the induction of autophagy^36^. To identify the signaling pathway by which autophagy is activated following DSGOST treatment, we examined the expression levels of p62 and LC3 conversion in a dose-dependent manner (Fig. 2a). DSGOST treatment increased the expression of LC3-II and decreased that of p62. To identify whether autophagy plays a role in DSGOST resistance in a time-dependent manner, we performed the western blot assay to detect the expression of autophagy markers (Fig. 2b). We monitored the accumulation of LC3B, Beclin-1, and ATG5 and the downregulation of p62 levels in gastric cancer cells. Furthermore, AGS and SNU-638 cells were transfected with GFP-LC3 plasmid and then treated with DSGOST (Fig. 2c). The presence of GFP-LC3 puncta was used as an indicator of autophagosome formation. GFP-LC3 puncta was approximately sixfold higher in DSGOST-treated AGS and SNU-638 cells than in DMSO-treated gastric cancer cells. To study whether DSGOST and TJ-38 induce autophagy, western blot assay was performed. Consequently, DSGOST and TJ38 induce autophagy activation by the accumulation of LC3B and ATG5 and the degradation of p62 in AGS and SNU-638 cells (Fig. 2d). To check whether DSGOST or TJ-38 in combination with cisplatin regulates autophagy, we checked the expression of autophagy markers using western blot assay (Fig. 2e). DSGOST and TJ-38 cause chemoresistance by inducing the accumulation of LC3B and ATG5 and degradation of p62, whereas cisplatin induces cell death by activating autophagy. Moreover, unlike cisplatin alone, the combination of cisplatin with DSGOST or TJ-38 induces chemoresistance by activating autophagy. Taken together, our findings suggest that DSGOST or TJ-38 in combination with cisplatin regulates chemoresistance via the activation of autophagy in gastric cancer.

### DSGOST regulates autophagy flux

**Fig. 3:**
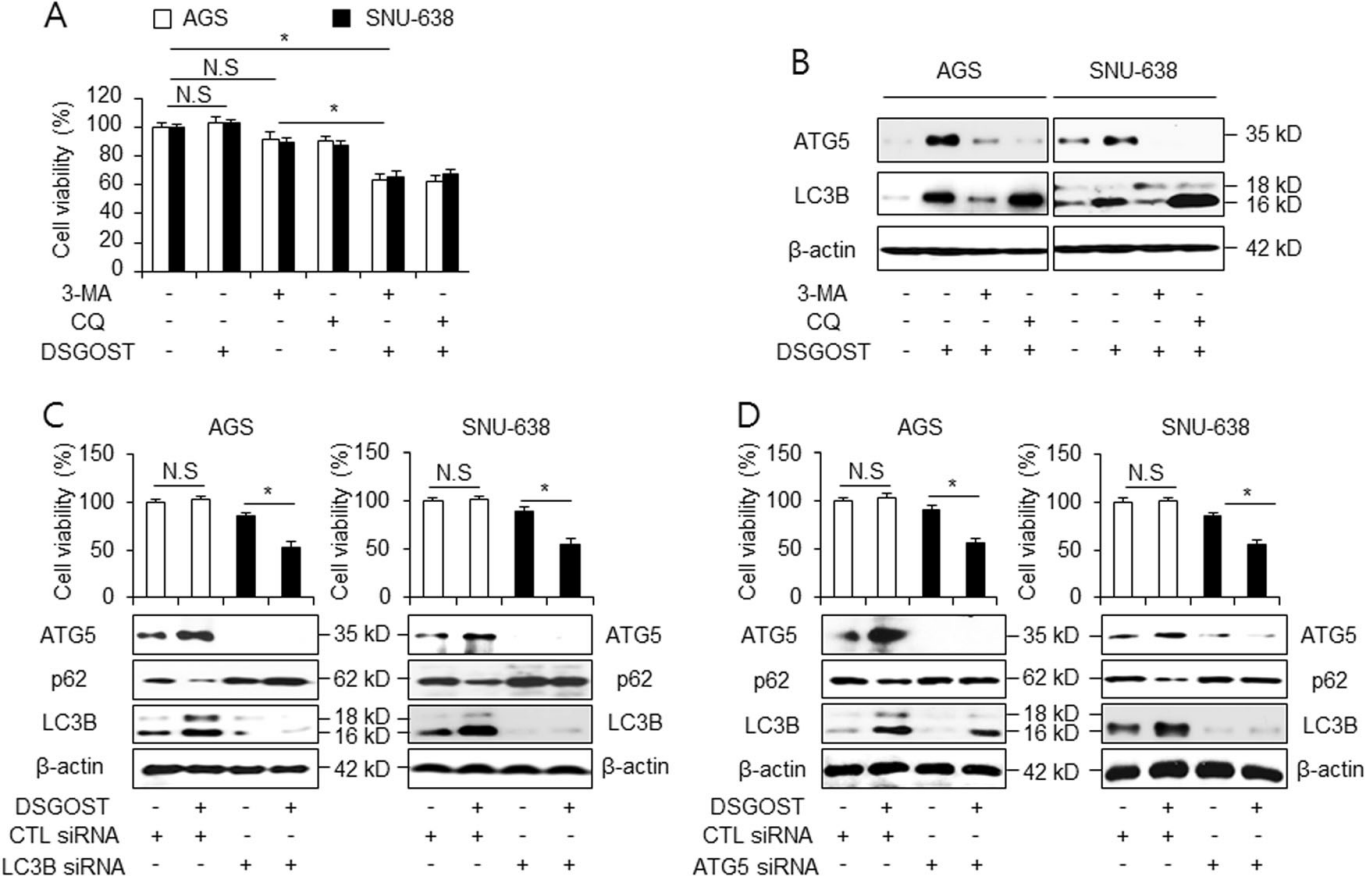
Autophagy inhibition regulates DSGOST-induced autophagic flux. **a** AGS and SNU-638 cells were treated with DSGOST (500 μg/mL, 24 h) in the absence or presence of the autophagy inhibitors 3-methylamine (3-MA, 5 mM) and chloroquine (CQ, 10 µM) for 24 h. Cell viability was performed and analyzed by WST-1 assay; *p < 0.05. **b** After AGS and SNU-638 cells were treated with DSGOST (500 μg/mL, 24 h) with or without 3-MA (5 mM) and CQ (10 µM) for 24 h, LC3B and ATG5 levels were examined using western blotting. **c** After transfection with control or LC3B siRNA in the presence and absence of DSGOST (500 μg/mL, 24 h), expression levels of LC3B, p62, and ATG5 were performed by western blot assay in AGS and SNU-638 cells. **d** AGS and SNU-638 cells were treated with DSGOST (500 μg/mL, 24 h) in the presence or absence of ATG5 siRNA

To further investigate the role of autophagy during DSGOST treatment, autophagy inhibitors, including 3-methyaldenine (3-MA) and chloroquine (CQ), were examined to arrest autophagy at early and late stages. We performed the WST-1 assay to determine whether 3-MA or CQ affected the DSGOST-induced cell viability in gastric cancer cells. The presence of the autophagy inhibitors did not affect cell viability in AGS and SNU-638 cells, whereas the treatment with 3-MA or CQ significantly decreased DSGOST-induced cell viability (Fig. 3a). To identify whether the induction of LC3B and ATG5 correlated with increased autophagic flux during DSGOST treatment, AGS and SNU-638 cells were treated with DSGOST in the presence or absence of 3-MA and CQ and western blot assay was performed. 3-MA reduced the activation of LC3B and ATG5 in DSGOST-mediated AGS and SNU-638 cells, whereas the treatment with CQ further increased the induction of LC3-II (Fig. 3b). CQ inhibits autophagy activation by blocking autophagolysosome formation^37^. This finding suggests that autophagy inhibition plays an important role in DSGOST-induced chemotherapy. To further confirm DSGOST-induced autophagy, AGS and SNU-638 cells were treated with DSGOST after transfection with LC3B- and ATG5-specific siRNA (Fig. 3c, d). The knockdown of LC3B and ATG5 significantly decreased cell viability and inhibited DSGOST-induced autophagy. This evidence suggests that autophagy inhibition could suppress DSGOST-caused autophagy activation.

### DSGOST induces cell death via autophagy inhibition

**Fig. 4:**
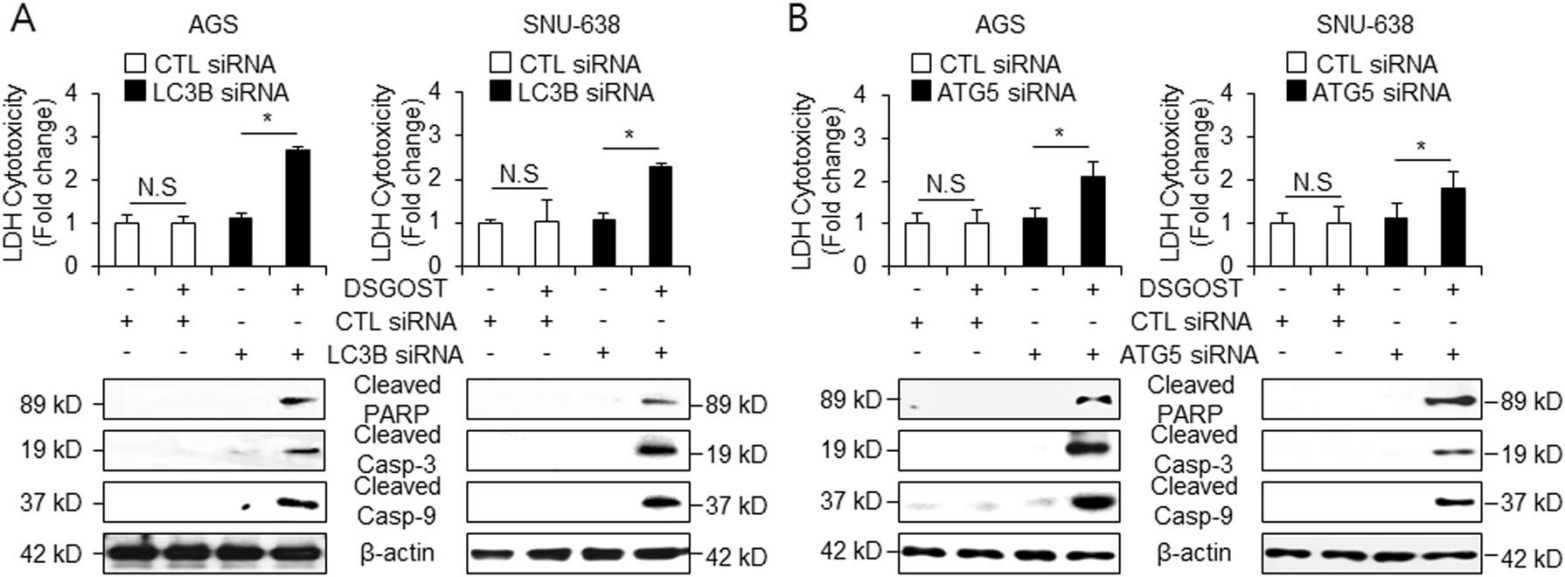
Autophagy inhibition enhances DSGOST-induced cell death. **a**, **b** After transfection by LC3B or ATG5 siRNA in AGS and SNU-638 cells, cell cytotoxicity was investigated with/without DSGOST (500 μg/mL, 24 h) using LDH assay, and cleaved caspase-3, caspase-9, and PARP were used to identify apoptosis by DSGOST in the presence or absence of LC3B or ATG5 siRNA using western blotting. β-actin was used as the protein loading control

To examine whether DSGOST induces cell death via autophagy inhibition, AGS and SNU-638 cells were transfected by LC3B and ATG5 siRNA. DSGOST-treated LDH release was significantly enhanced in DSGOST-treated LC3B and ATG5 siRNA transfected cells, whereas no increase was observed in control cells (Fig. 4a, b). We investigated whether autophagy inhibition affects the expression of pro-apoptotic factors using western blot assays. As expected, compared with DSGOST treatment in control cells, DSGOST treatment in LC3B or ATG5 knockdown cells resulted in a significant increase of cleaved caspase-3 and -9 expression and PARP cleavage (Fig. 4a, b). Taken together, these results suggest that autophagy inhibition mediates DSGOST-induced cell death in gastric cancer.

### DSGOST leads to dissociation of Beclin-1−Bcl2 complex

**Fig. 5:**
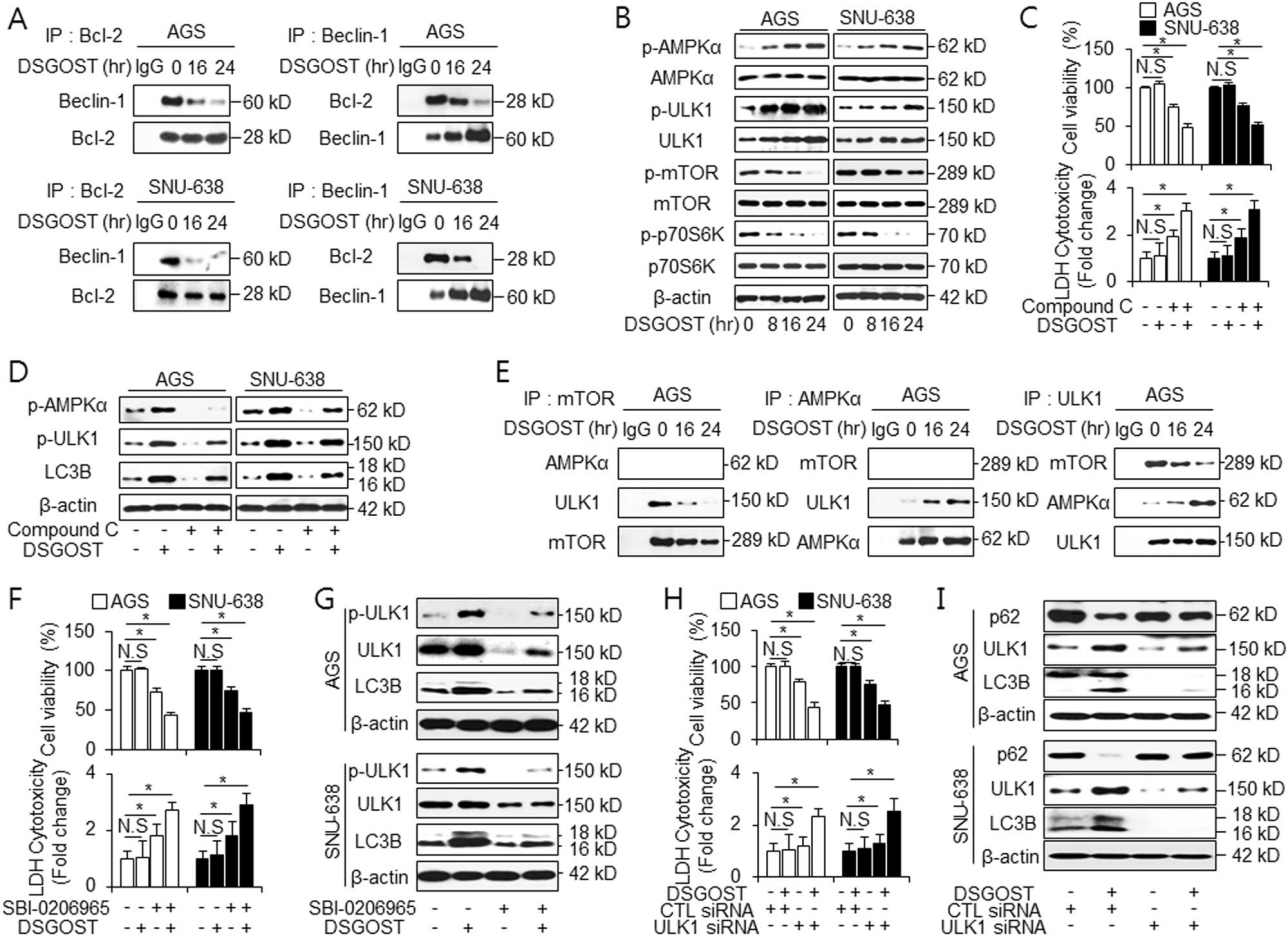
DSGOST dissociates between the Beclin-1 and Bcl-2 complex and activates AMPK/ULK1 pathway in gastric cancer cells. **a** AGS and SNU-638 cells were treated with DSGOST (500 μg/mL) for 16 and 24 h. Bcl-2 was immunoprecipitated in AGS and SNU-638 cells, and the immunoprecipitated proteins were subjected to western blotting. Beclin-1 was detected in immunoprecipitates prepared with anti-Bcl-2 antibody by immunoprecipitation. Bcl-2 was also monitored in immunoprecipitates prepared with anti-Beclin-1 antibody by immunoprecipitation. **b** AGS and SNU-638 cells were treated with DSGOST (500 μg/mL) in a time-dependent manner (0, 8, 16, and 24 h). After DSGOST treatment, cell lysates were loaded to western blot assay and detected antibodies targeting p-AMPKα (Thr172), AMPKα, p-ULK1 (Ser757), ULK1, p-mTOR (Ser2448), mTOR, p-p70S6K (Thr389), and p70S6K for autophagy induction. β-actin was used as the protein loading control. **c**, **d** After AGS and SNU-638 cells were treated with DSGOST (500 μg/mL, 24 h) in the absence or presence of Compound C (Sigma, 2 µM), cell viability and LDH release were analyzed by WST-1 and LDH assays. Cell lysates were analyzed for the level of indicated proteins. **e** AGS cells were treated with DSGOST (500 μg/mL) for 16 and 24 h. AMPKα, mTOR, and ULK1 were immunoprecipitated in DSGOST-treated AGS cells and the immunoprecipitated proteins were subjected to western blotting using mTOR, AMPKα, and ULK1 antibodies, respectively. **f**, **g** Cell viability, LDH assay, and western blotting for LC3B, p62, and ULK1 were performed in gastric cancer cells treated with DSGOST (500 μg/mL) in the presence or absence of SBI-0206965 (Sigma, 10 µM) for 24 h. **h**, **i** After AGS and SNU-638 cells were treated with DSGOST (500 μg/mL, 24 h) in the absence or presence of control and ULK1 siRNA, cell viability and LDH assays were performed. Western blotting was carried out using antibodies for LC3B, p62, and ULK1. β-actin was used as the protein loading control. *p < 0.05

The interaction between Beclin-1 and Bcl-2 reportedly alters autophagy function of Beclin-1^38^. To confirm whether DSGOST can interfere with the interaction between Beclin-1 and Bcl-2, we examined the co-immunoprecipitation of Beclin-1 and Bcl-2 in DSGOST-mediated gastric cancer cells (Fig. 5a). First, Bcl-2 and Beclin-1 antibodies were immunoprecipitated in DSGOST-treated gastric cancer cells. In control cells, an interaction between Bcl-2 and Beclin-1 was detected in the immunoprecipitated fractions by co-immunoprecipitation, indicating an interaction between Bcl-2 and Beclin-1. However, DSGOST treatment in these cells induced a time-dependent disruption of this complex.

### DSGOST induces resistance via activation of the AMPKα/ULK1 pathway

A previous study identified that AMP-activated protein kinase α (AMPKα) activates autophagy by activating serine/threonine kinases 1 (ULK1) and inhibiting the mammalian target of rapamycin (mTOR)/ribosomal protein S6 kinase p70S6K (p70S6K) signaling^39^. Therefore, using western blotting, we investigated whether the AMPKα/ULK1 and mTOR/p70S6K pathways were involved in DSGOST-induced autophagy in gastric cancer cells. DSGOST reduced in the phosphorylation levels of mTOR in a time-dependent manner, whereas total mTOR levels were not affected by DSGOST treatment (Fig. 5b). In addition, DSGOST induced a reduction in p70S6K phosphorylation, indicating its potent inhibitory effect by DSGOST on mTOR/p70S6K signaling (Fig. 5b). AMPK, by interacting with ULK1, is involved in the initiation of autophagosome formation^40^. We explored the activation of AMPKα and ULK1 in DSGOST-mediated gastric cancer cells. AMPKα and ULK1 could be activated by DSGOST in a time-dependent manner in AGS and SNU-638 cells (Fig. 5b). As autophagy promotes cell survival, our study investigated whether the inhibition of AMPK augmented DSGOST cytotoxicity. As expected, Compound C, a well-known inhibitor of AMPK, mediated DSGOST-induced cell death, which was indicated by a decrease in cell viability and an increase in LDH release in the DSGOST + Compound C group compared with the other groups (Fig. 5c). The inhibition of AMPKα resulted in lower expression levels of p-AMPKα, p-ULK1, and LC3-II in the DSGOST + Compound C group than in the DSGOST group, indicating that AMPKα inhibition interrupted DSGOST-mediated autophagy (Fig. 5d).

To further determine how DSGOST regulates autophagy signaling, AGS cells were exposed to DSGOST in a time-dependent manner, and the immunoprecipitated fractions were isolated using antibodies, such as mTOR, AMPKα, and ULK1 (Fig. 5e). In this study, mTOR did not interact with AMPKα in DSGOST-mediated AGS cells. However, an interaction between mTOR and ULK1 was observed in immunoprecipitated fractions and DSGOST caused a time-dependent disruption of this interaction (Fig. 5e). AMPKα did not interact with ULK1 in DMSO-treated AGS cells. Interestingly, DSGOST induced a time-dependent interaction of AMPKα and ULK1 (Fig. 5e). Our finding was identified by immunoprecipitation using ULK1 antibody. Taken together, mTOR and ULK1 were found to interact during DMSO treatment, but DSGOST caused the dissociation of mTOR and ULK1 and induced the interaction of AMPKα and ULK1 (Fig. 5e). These findings suggest that the activation of the AMPKα/ULK1 pathway is an important event in DSGOST-induced pro-survival autophagy.

Next, we treated with either the presence or the absence of SBI-0206965, which is a small molecule inhibitor for ULK1 in DSGOST-treated gastric cancer cells. Consistently, our results showed that DSGOST + SBI-0206965 treatment induced a reduction of cell viability and an increase of LDH release compared with DSGOST treatment (Fig. 5f). The pharmacological inhibition of ULK1 resulted in lower expression levels of p-ULK1 and LC3-II in the DSGOST + SBI-0206965 group compared with the DSGOST group, indicating that the inhibition of ULK1 interrupted DSGOST-mediated autophagy (Fig. 5g). To gain more insight into the role of ULK1 in regulating DSGOST-induced autophagy, AGS and SNU-638 cells were transfected with ULK1 siRNA. The knockdown of ULK1 induced a decrease of cell viability and an increase of LDH release in DSGOST-treated gastric cancer cells (Fig. 5h). Furthermore, the ULK1 knockdown cells decreased LC3B level and slightly reduced p62 level in DSGOST-treated gastric cancer cells compared with the control cells (Fig. 5i). These findings indicated that ULK1 inhibition could effectively overcompensate for DSGOST resistance and enhance the sensitivity of gastric cancer cells to DSGOST-mediated cell death.

### DSGOST induces autophagy and EMT in gastric cancer

**Fig. 6:**
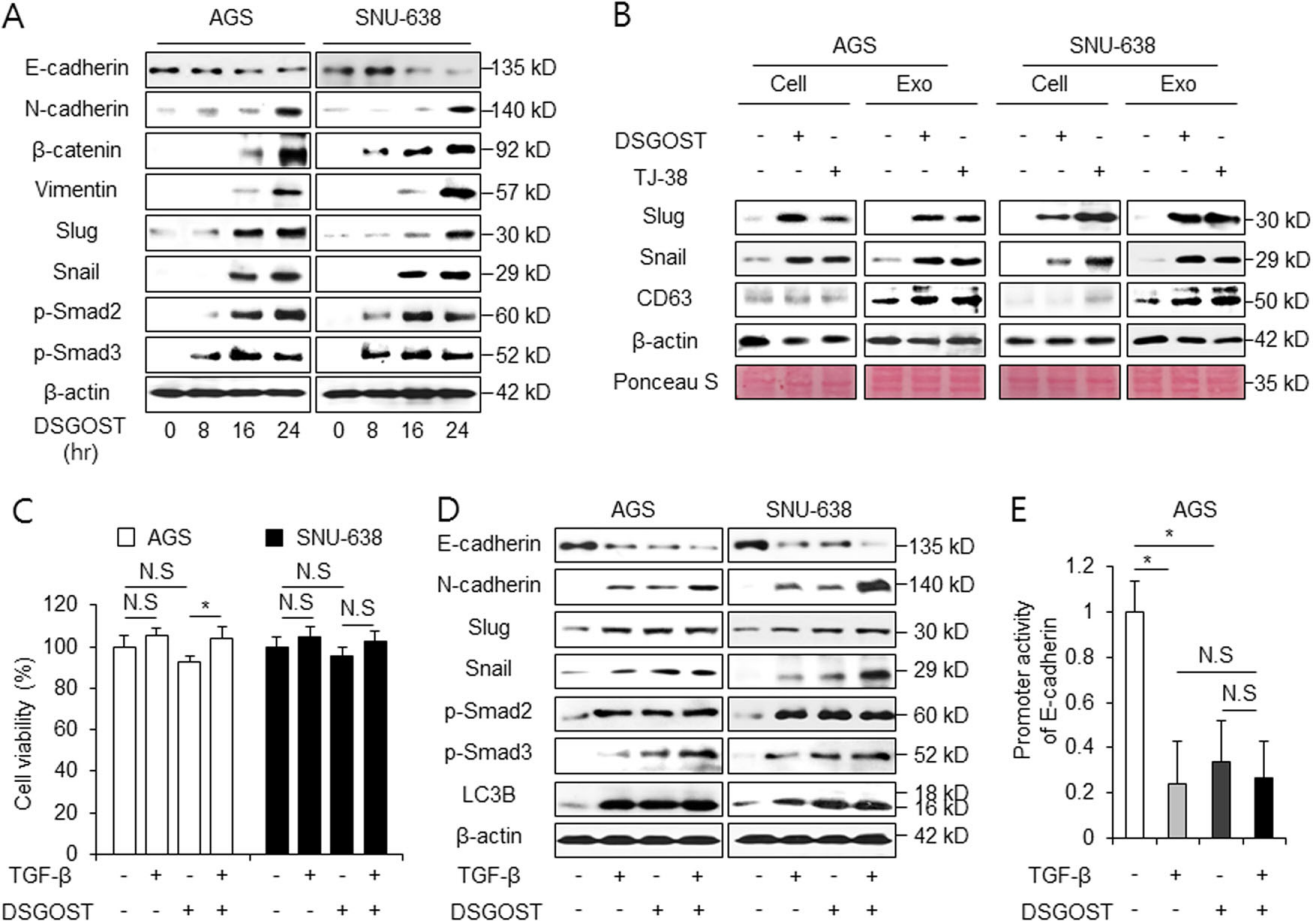
DSGOST induces EMT in gastric cancer. **a** AGS and SNU-638 cells were treated with DSGOST (500 μg/mL) in a time-dependent manner (0, 8, 16, and 24 h). After DSGOST treatment, extracted total proteins were subjected to western blotting using antibodies for EMT markers, such as E-cadherin, N-cadherin, β-catenin, vimentin, Slug, Snail, and Smad signaling, including p-Smad2 and p-Smad3. **b** AGS and SNU-638 cells were treated with DSGOST or TJ-38 and exosomes were collected from cell supernatants. Exosomes and cell lysates were quantified by Ponceau S staining. Exosomes were also examined by western blotting using the exosome marker CD63 and EMT markers, such as Snail and Slug. **c**, **d** Cell viability and western blotting analysis of EMT markers and Smad signaling in AGS and SNU-638 cells after TGF-β1 (5 ng/mL, 24 h) and DSGOST (500 μg/mL, 24 h) treatment; *p < 0.05. **e** AGS cells were transfected with a reporter luciferase vector containing an E-cadherin promoter (−368~+51), and were treated with TGF-β1 (5 ng/mL, 24 h) or/and DSGOST (500 μg/mL, 24 h). The luciferase activity was normalized to the activity of Renilla luciferase; *p < 0.05

Increasing evidences suggest that increasing EMT in cancer plays a powerful role in enabling malignancy features^41^. Furthermore, protective autophagy regulates chemoresistance and EMT via TGF-β/Smad signaling^42^. To determine whether DSGOST promotes EMT in gastric cancer cells, the exposure of these cells to DSGOST was studied in a time-dependent manner, and EMT markers, such as E-cadherin, N-cadherin, β-catenin, vimentin, Slug, Snail, p-Smad2, and p-Smad3, were examined. We observed that E-cadherin decreased in DSGOST-treated gastric cancer cells, whereas all other accumulated in DSGOST-treated cells (Fig. 6a). These data indicate that DSGOST-induced autophagy triggers EMT in gastric cancer cells. A recent report suggested that cancer-derived exosomes are an important mediator of autophagy and EMT signaling^43^. Robust evidence also indicates that autophagy plays a key role in chemoresistance, exosome release, and EMT in tumor cells^44^. Western blotting revealed that exosomes purified from DSGOST or TJ-38-treated gastric cancer cells were highly positive for the exosome markers, such as CD63 (Fig. 6b). Our results suggest that DSGOST- or TJ-38-treated cell lysates and exosomes induce the upregulation of Slug and Snail (Fig. 6b). Therefore, these findings suggest that DSGOST or TJ-38-induced autophagy regulates EMT signaling via exosome release.

TGFβ is the core EMT transcription factor and autophagy promotes TGFβ-induced EMT^45^. We investigated whether DSGOST regulates EMT in TGFβ-treated gastric cancer cells. Similar to DMSO-treated cells, cell viability of DSGOST-mediated gastric cancer cells was not affected by TGFβ treatment (Fig. 6c). To explore whether DSGOST in combination with TGFβ induces autophagy and EMT in gastric cancer cells, we examined LC3B and EMT markers. Western blot results demonstrated a significant decrease of E-cadherin and an increase of LC3B, N-cadherin, Slug, Snail, p-Smad2, and p-Smad3 in DSGOST and TGFβ-treated AGS and SNU-638 cells (Fig. 6d). We constructed a luciferase reporter driven by the E-cadherin promoter. Luciferase assay analyzed the activity of the E-cadherin promoter in TGFβ and DSGOST-treated AGS cells. These cells showed significantly decreased activity of this promoter, indicating EMT activation (Fig. 6e). These findings suggest that DSGOST promotes autophagy and EMT in TGFβ-treated gastric cancer cells.

### Autophagy inhibition blocks DSGOST-caused EMT

**Fig. 7:**
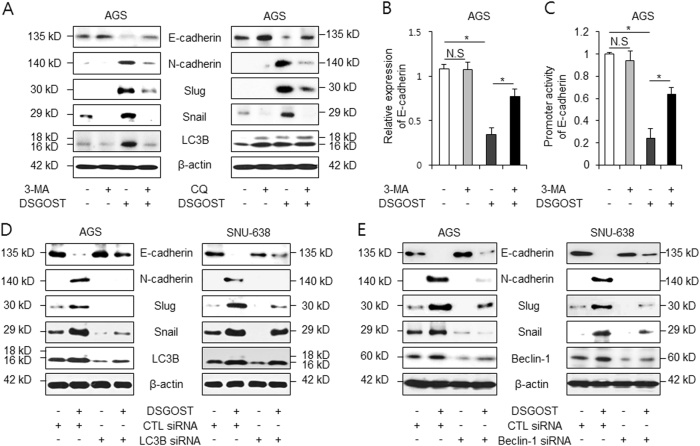
Autophagy inhibition suppresses DSGOST-induced EMT phenomenon in gastric cancer. **a** AGS cells were treated with DSGOST (500 μg/mL, 24 h) in the absence or presence of 3-MA (5 mM, left) and CQ (10 µM, right). Extracted proteins were subjected to western blotting using antibody for EMT markers, such as E-cadherin, N-cadherin, vimentin, Slug, Snail, and LC3B. **b** Real-time RT-PCR was performed using primers for E-cadherin and β-actin in DSGOST (500 μg/mL, 24 h)- and/or 3-MA (5 mM, 24 h)-treated AGS cells. **c** AGS cells were transfected with a reporter luciferase vector (pGL2) containing E-cadherin promoter (−368~+51) and treated with DSGOST (500 μg/mL, 24 h) and/or 3-MA (5 mM, 24 h). *p < 0.05. **d**, **e** After transfection by LC3B or Beclin-1 siRNA in AGS and SNU-638 cells, these cells were treated with/without DSGOST (500 μg/mL, 24 h). Extracted proteins were subjected to western blotting using antibodies for EMT markers, including E-cadherin, N-cadherin, Slug, Snail, and LC3B. β-actin was used as the protein loading control

To characterize the relationship between DSGOST-induced autophagy and EMT, DSGOST was treated with 3-MA or CQ in AGS cells. As a result, the expression of LC3B, N-cadherin, Slug, and Snail was dramatically decreased compared with the expression observed with the treatment with DSGOST alone (Fig. 7a). Transcriptional regulation of E-cadherin regulates EMT, and Snail is a repressor of E-cadherin in tumor cells^46,47^. To elucidate the effect of autophagy on the EMT process, real-time RT-PCR was performed. We identified that E-cadherin expression was not changed by 3-MA in AGS cells (Fig. 7b). However, E-cadherin expression had higher mRNA levels in DSGOST plus 3-MA-treated AGS cells than in DSGOST-treated cells. To confirm the role of E-cadherin promoter activity in DSGOST plus 3-MA-treated AGS cells, we performed promoter luciferase reporter assays. We found that the E-cadherin promoter had a higher luciferase reporter activity in DSGOST plus 3-MA-treated AGS cells than in DSGOST-treated cells (Fig. 7c). To further clarify the association between autophagy and EMT, gastric cancer cells were transfected by LC3B and Beclin-1 siRNAs and treated with DSGOST. These experiments showed lower expression of LC3B, Beclin-1, N-cadherin, Slug, and Snail than control cells (Fig. 7d, e). Taken together, these findings suggest that autophagy inhibition regulates DSGOST-induced EMT signaling in gastric cancer.

## Discussion

Currently, there is now emerging interest and scientific evidence in the use of DSGOST as an anticancer therapeutic. However, the mechanisms underlying chemoresistance, EMT, and exosome in DSGOST-induced autophagy remain poorly understood. In this study, we found that DSGOST regulates resistance via prosurvival autophagy in gastric cancer and induces the expression of EMT markers in exosomes and cell lysates. We identified that autophagy inhibition mediates DSGOST-induced cell death and suppresses EMT. Our study has showed that autophagy plays an important role in cell survival and death of DSGOST-treated gastric cancer cells.

Previous studies have reported on the use of DSGOST in the therapy of vascular disease, such as RP^4,48^. RP is caused by anticancer agents, such as bleomycin and cisplatin and anticancer therapy has several early or late side effects, including RP and hypersensitivity syndrome^49,50^. In cancer chemotherapy, it is of utmost importance to prevent the side effects of anticancer agents. Our robust evidence based on a clinical trial of DSGOST performed on Korean female patients provided evidence of its potential for use as a powerful drug for RP therapy^51^. We hypothesized that DSGOST would help resolve RP that may occur due to chemotherapy using anticancer drug such as cisplatin. Previous results identified that DSGOST inhibited VEGF-induced tumor angiogenesis in endothelial cells and suggested it as a noble strategy for some relief during cancer chemotherapy^17^. We found that DSGOST and TJ-38-induced autophagy significantly reduced cisplatin-mediated cell death. Therefore, these findings suggest that a prosurvival autophagic response was associated with chemoresistance in DSGOST plus cisplatin-treated gastric cancer cells. The success of anticancer therapy is frequently limited by drug alone or multidrug resistance^52^. Increasing studies have shown that autophagy plays a powerful role in chemoresistance^53^. Some recent reports suggest that protective autophagy confers chemoresistance to anticancer drugs, including cisplatin, in gastric cancer^54^. In this study, DSGOST and TJ-38 induced autophagy by enhancing LC3-II and ATG5 and reducing p62 in gastric cancer cells, thus contributing to chemoresistance.

DSGOST dissociated the Beclin-1−Bcl-2 complex to initiate autophagy and activated AMPK/ULK1 signaling. When AGS cells were treated with DSGOST, AMPK interacted with ULK1 to regulate DSGOST-mediated autophagy and inhibited the mTOR/p70S6K pathway, which inactivates autophagy. Consistent with this finding, we found that AMPK activation after DSGOST treatment induces autophagy through ULK1 phosphorylation in gastric cancer cells. The inhibition of AMPK and ULK1 decreased DSGOST-induced autophagy in gastric cancer cells, thus leading to an increase in DSGOST-caused cell death. According to a recent report, protective autophagy was correlated with acquired drug resistance, and the inhibition of autophagy was suggested as a noble therapeutic strategy in cancer^55^. O’Donovan et al. also reported that 5-fluorouracil (5-FU) and cisplatin-resistant cell lines show a higher autophagy activation response than chemo-sensitive cell lines; further, autophagy inhibition has a chemotherapeutic effect in drug-resistant cancer cells^31^. Taken together, our data identified that DSGOST regulates resistance via autophagy induction in gastric cancer cells, whereas autophagy inhibition induces cell death in DSGOST-treated gastric cancer cells.

Starvation-mediated autophagy is critical for cellular invasion via EMT activation in hepatocellular carcinoma cells, and TGFβ1 stimulates EMT and autophagy via the activation of Smad signaling in cancer cells^56^. Protective autophagy promotes EMT process, and EMT also promotes drug resistance^57^. Our findings indicate that DSGOST treatment regulates EMT by reducing E-cadherin and increasing of mesenchymal markers including N-cadherin and vimentin in gastric cancer cells. In addition, EMT regulators, such as Snail and Slug, were upregulated in lysates and exosomes derived from DSGOST-treated gastric cancer cells, and TGFβ1 plus DSGOST treatment induced EMT signaling via phosphorylation of Smad2 and Smad3. We suggest that the DSGOST-induced EMT activation may contribute to resistance via autophagy activation. The inhibition of DSGOST-mediated autophagy blocked the acquisition of the EMT phenotype and induced DSGOST-stimulated cell death. These findings suggest that autophagy inhibition is a potential therapeutic strategy in DSGOST-treated gastric cancer.

Exosomes are small membrane vesicles derived from the endosomal system and also contribute to EMT in cancer^58^. A recent report indicates that cells may communicate with neighbors via exosome secretion, and this has recently gained attention for understanding chemoresistance mechanisms^59^. A recent study reported that exosomes derived from docetaxel-resistant breast cancer cells reduced the antiproliferative effects^60^. Autophagy is a part of the endolysosomal membrane system and is linked to exosome production^61^. Furthermore, when exosomes derived from gefitinib-induced PC9 cells were exposed to cisplatin-treated cells, gefitinib-treated exosomes significantly decreased the antitumor effects of cisplatin by activating autophagy^62^. Exosomes derived from DSGOST or TJ-38-treated gastric cancer cells were highly positive for the exosomal marker protein CD63 and the upregulation of Slug and Snail was found within these exosomes. These results mean that DSGOST-induced autophagy regulates resistance via EMT process and exosome release.

To conclude, for the first time, we demonstrated that DSGOST in combination with cisplatin suggests a new therapeutic strategy for anticancer therapy and may inhibit the chemotherapy adverse effects of RP. Furthermore, our findings propose new insights into the molecular mechanisms underlying DSGOST-induced resistance via autophagy activation in gastric cancer cells.

## Materials and methods

### DSGOST extraction

DSGOST was extracted as previously described^17^. This herbal formula was originally designed for cancer therapy. The nine ingredients and their amounts (g) were prepared as follow: 1 g of *Angelica gigas*, 1 g of *Cinnamomum cassia* Blume, 1 g of *Paeonia lactiflora* Pallas, 1 g of Akebia root, 0.67 g of Asarum, 0.67 g of *Glycyrrhiza uralensis* Fischer, 1.67 g of *Zizyphus jujuba* var. *inermis* Rehder, 0.67 g of Evodia fruit, and 1.33 g of *Zingiber officinale* Rosc. The mixtures were obtained by Han-Poong Pharm Co. Ltd (Jeonjoo, Repubic of Korea). Herbal medicines were mixed together, soaked in water, and extracted by 100 °C treatment for 2 h. The extract was then filtered, evaporated, and lyophilized to make DSGOST powder. This was stored at −80 °C until use.

### Cell culture

The human gastric cancer cell lines (AGS, SNU-216, NCI-N87, SNU-638, SNU-668, and MKN-74) were purchased from the Korean Cell Line Bank (Cancer Research Center, Seoul National University, Seoul, Korea). Cells were cultured in RPMI1640 medium (Welgene) supplemented with 10% fetal bovine serum (JR Scientific) and 100 μg/mL antibiotics (100 U/mL penicillin and 100 μg/mL streptomycin, Welgene) in a 5% CO_2_ humidified incubator at 37 °C.

### Cell viability assay

Cell viability was determined by the WST-1 assay. It was performed according to the manufacturer’s instructions (Roche). Cells were seeded to each well of a 96-well plate (1×10^4^ cells/well) and incubated for 24 h (5% CO_2_ humidified incubator at 37 °C). On day 1 after cell seeding, cells were treated with various doses (100, 300, and 500 µg/mL) of DSGOST and for various times (4, 16, and 24 h). The autophagy inhibitors 3-MA (5 mM, Sigma) and CQ (20 µM, Sigma) were added to the RPMI1640 medium for 24 h. Gastric cancer cells were treated with TJ-38 (Tsumura & Co., Japan) in a dose-dependent manner (100, 300, and 500 µg/mL). These cells was treated with cisplatin (5 µM, Sigma), SBI-0206965 (5 µM, Sigma), and TGF-β1 (5 ng/mL, R&D Systems). Then, 10 μL of WST-1 reagent was added in a 96-well plate, and the conversion of the WST-1 reagent into chromogenic formazan was evaluated with a spectrophotometer (Molecular devices, USA) after 1 h.

### LDH assay

AGS and SNU-638 cells (1×10^4^ cells/well) were seeded into a 96-well plate with growth medium. To determine the LDH (Thermo Scientific Pierce) activity in supernatants, 100 μL of the reaction mixture was added, and incubation for 30 min was performed in a dark room. The LDH activity measured the absorbance of the samples at 490 or 492 nm using ELISA reader.

### Transfection

AGS and SNU-638 cells (2×10^5^ cells/well) in a six-well plate were transfected with these double-stranded siRNAs (30 nmol/mL), including LC3B (Sigma), ULK1, Beclin-1, and ATG5 (Santacruz) for 24 h by the Lipofectamine 2000 reagent (Invitrogen) method according to the manufacturer’s protocol and recovered in RPMI1640 medium (Welgene) containing 10% fetal bovine serum (JR Scientific) and 100 μg/mL antibiotics (100 U/mL penicillin and 100 μg/mL streptomyhcin, Welgene) for 24 h. After recovery, viable cells were calculated by WST-1.

### Isolation of total RNA and protein

Total RNA from gastric cancer cells (2×10^6^ cells/well) in 100 mm cell culture dish was prepared using Trizol reagent according to the manufacturer’s protocols (Invitrogen). Protein sampling was collected in RIPA buffer (Biosesang) containing a protease inhibitor cocktail (Sigma) on ice for 30 min and were passed through an 18-gauge needle, and spin down. The supernatant was analyzed for protein content using the BCA method (Thermo Scientific).

### Real-time PCR and western analysis

The E-cadherin expression level was measured by real-time PCR using cDNA synthesized from 5 μg of total RNA and a reverse transcription kit (Promega). Reactions were performed in triplicate for each sample using an ABI Power SYBR green PCR Master Mix (Applied Biosystems) with E-cadherin-specific primers (5′-GAACGCATTGCCACATACAC-3′ (sense) and 5′-GAATTCGGGCTTGTTGTCAT-3′ (antisense)) on a Roche LightCycler 96 (Roche). RNA quantify was normalized to β-actin primers (5′-AAGGCCAAC CGCGAGAAGAT-3′ (sense) and 5′-TGATGACCTGGCCGTCAGG-3′ (antisense)), and gene expression was quantified according to the 2^−ΔCt^ method. To conduct the western blot assay, gastric cancer cells were solubilized in radioimmunoprecipitation assay (RIPA) lysis buffer (50 mM/L Tris-HCl (pH 7.4), 150 mM/L NaCl, 1% NP40, 0.25% sodium deoxycholate, 1 mM/L phenylmethylsulfonylfluoride, 1 mM/L sodium orthovanadate, 1× sigma protease inhibitor cocktail) and protein was measured using a standard bicinchoninic acid assay. Equal amounts of protein (20 μg) were size-fractionated by 8~15% SDS-PAGE and then transferred onto an NC membrane (Millipore Corporation). Membranes were blocked by incubation for 30 min with 5% skim milk/PBS-T, and incubated overnight at 4 °C with primary antibodies diluted in 1× PBST buffer. The following primary antibodies were used: β-actin, Bcl-2, Beclin-1, ULK1, and Atg5 (Santa Cruz, 1:1000); LC3B and p62 (Sigma, 1:1000); CD63 (Abcam, 1:1000); cleaved caspase-3, caspase-9, -PARP, p62, AMPKα, p-AMPKα (Thr172), mTOR, p-mTOR (Ser2448), p70S6K, p-p70S6K (Thr389), p-ULK1 (Ser757), E-cadherin, N-cadherin, β-catenin, vimentin, p-Smad2, p-Smad3, Slug, and Snail (Cell Signaling, 1:1000). The membranes were washed three times with PBST buffer. The secondary antibody was diluted in PBST or TBST buffer and was added for 40 min at room temperature. The following secondary antibodies were used: anti-rabbit IgG HRP-linked antibody and anti-mouse IgG HRP-linked antibody (KPL, 1:6000). The membranes were washed six times with PBST buffer for 1 h. The blots were visualized by Western Chemiluminescent HRP Substrate (Millipore).

### Quantification of pEGFP-LC3 puncta

AGS and SNU-638 cells (2×10^5^ cells/well) in a six-well plate were transfected with pEGFP-LC3 using Lipofectamine 2000 (Invitrogen), and then treated with 500 μg/mL DSGOST for 8 h. A pEGFP-LC3B-positive punctate pattern was observed by confocal microscopy. Confocal microscopy was conducted using a ZEISS LSM5 PASCAL confocal microscope with 405- and 488-nm excitation lasers.

### Luciferase reporter assay

To evaluate the E-cadherin promoter activity, AGS cells (2×10^5^ cell/well) were seeded in a six-well plate and transfected with 2.5 μg of pGL2 luciferase vector (Promega) and reporter luciferase vector containing an E-cadherin promoter (-368~+51) after 24 h using Lipofectamine 2000 agent (Invitrogen). Luciferase activity was measured 36 h after transfection in three independent cultures using a dual-luciferase reporter assay kit (Promega) on Molecular Devices Filter Max F3 (Molecular Devices). The luciferase activity was normalized to activity of the Renilla luciferase.

### Immunoprecipitation (IP)

We extracted cell lysates from AGS and SNU-638 cells (2×10^6^/well) on a 100 mm cell culture plate in a buffer containing 50 mM Tris-HCl, pH 7.5, 250 mM NaCl, 5 mM EDTA, 0.5% (v/v) NP-40 and a protease inhibitor cocktail (Sigma). We incubated anti-Bcl-2 (Santa Cruz), anti-Beclin-1 (Santa Cruz), mTOR (Santa Cruz), AMPK (Cell Signaling), and ULK1 (Cell Signaling) with lysate at 4 ℃ for 16 h. We used the protein A/G PLUS agarose (Santa Cruz) to pull down immunocomplexes. We washed precipitates three times with a solution containing 50 mM Tris-HCl, pH 7.5, 250 mM NaCl, 5 mM EDTA, and 0.5% (v/v) NP-40.

### Exosome isolation

Exosomes were obtained from the supernatant of untreated and DSGOST (0, 300, and 500 μg/mL)-treated AGS and SNU-638 cells according to the manufacturer’s protocols (Total Exosome Isolation Reagent (from cell culture media), Thermo Fisher Scientific). Protein concentration was measured using the BCA method (Thermo Scientific). Samples with equal protein loading (15 μg) were also quantified by Ponceau S staining and were subjected to western blot assay. Positive exosomes were detected by the exosome marker, CD63 (Abcam).

### Statistical analysis

All results were confirmed in at least three independent experiments; Student’s *t* tests were used for comparisons of means of quantitative data between groups and *p* < 0.05 was considered statistically significant.
